# Prognostic Signature, Immune Features, and Therapeutic Responses of a Novel Ubiquitination-Related Gene Signature in Lung Adenocarcinoma

**DOI:** 10.1155/2022/2524649

**Published:** 2022-08-16

**Authors:** Muge Xu, Jiening Gong

**Affiliations:** School of Chinese Medicine, School of Integrated Chinese and Western Medicine, Nanjing University of Chinese Medicine, Nanjing 210023, China

## Abstract

Growing studies have implicated the association of ubiquitination-related genes (UbRGs) with the cancer progression and the long-term survival of patients. However, the prognostic values of UbRGs in lung adenocarcinoma (LUAD) have not been investigated. Our study aimed to establish a ubiquitination-related model for prognosis prediction and internal mechanism investigation. The transcriptome expression profiles and corresponding clinical information of LUAD were obtained from TCGA and GEO datasets. Differentially expressed genes (DEGs) were screened between LUAD specimens and nontumor specimens. Kaplan–Meier analysis and univariate assays were carried out on DEGs to preliminarily screen survival-related UbRGs. Then, the LASSO Cox regression model was applied to develop a multigene signature, which was then demonstrated in two GEO datasets by the use of Kaplan-Meier, ROC, and Cox analyses. We estimated the immune cell infiltration in tumor microenvironment via CIBERSORT and immunotherapy response through the TIDE algorithm. In this study, a total of 71 ubiquitination-related DEGs were identified. Nine UbRGs, including TUBA4A, TRIM2, PLK1, ARRB1, TRIM58, PLK1, ARRB1, CCNB1, TRIM6, PTTG1, and CCT2, were included to establish a risk model, which was validated in TCGA and GEO datasets. The multivariate assays demonstrated that the 9-UbRGs signature was a robust independent prognostic factor in the overall survival of LUAD patients. The abundance of CD8 T cells, activated CD4 T memory cells, resting NK cells and macrophages was higher in the high-risk group, and the TMB of high-risk group was statistically higher than the low-risk group. Multiple drugs approved by FAD, targeting UbRGs, were available for the treatment of LUAD. Overall, we identified a nine ubiquitination-related gene signature, and the signature may be applied to be a potential biomarker for CD8 T cells response and clinical responses to immune checkpoint inhibitors for LUAD.

## 1. Introduction

Lung cancer is a fatal malignancy and one of the primary causes of tumor-related mortality, with 2,205,000 new cases and 1,790,000 deaths in 2020 worldwide [1]. Among different types of lung cancers, non-small-cell lung cancer (NSCLC) is the most prevalent accounting for around 85% of all lung cancers [2, 3], of which, lung adenocarcinoma (LUAD) is the most common form. In recent years, significant advancement in treatment has been made, most notably in the field of targeted therapy, and immunotherapy has developed as a new therapy [4, 5]. However, these two approaches are only able to help a small percentage of LUAD subtypes, and the overall survival rate of LUAD patients is still low [6, 7]. Several research studies have shown that LUAD had a heterogeneous condition with unique genetic and transcriptome traits among individuals, and it is still difficult to anticipate how an individual would fare with LUAD [8]. In light of this, it is absolutely necessary to locate reliable diagnostic indicators, treatment targets, and prognosis factors.

In response to physiological signals, biological processes known as dynamic modulation of proteins and posttranslational modification of proteins take place [9, 10]. Both of these processes are closely controlled. One example of this type of dynamic modulation is the process of ubiquitination, which tags proteins as candidates for breakdown by the proteasome [11]. This process also changes the proteins' location, affecting their activity and either promoting or inhibiting protein interactions [12]. Therefore, ubiquitination is essential for a wide variety of physiological activities, such as cell survival, differentiation, and innate and adaptive immune responses [13, 14]. An abnormal control of inflammatory pathways by ubiquitin has been documented in a variety of diseases, including malignancies and autoinflammatory diseases, and there is growing evidence to support this hypothesis [15, 16]. Therefore, addressing defective parts of the ubiquitin system as a potential treatment for inflammatory diseases, malignancies, and infectious diseases is an approach that holds a lot of promise. Furthermore, the creation of targetable molecular subtypes and risk stratification tools by making use of ubiquitination-related genes (UbRGs) has a great deal of promise [17, 18]. On the other hand, as far as we are aware, there are no studies done in the past that investigated whether there was a correlation between ubiquitination and the prognostic evaluation and molecular subtypes of LUAD.

In light of the fact that the ubiquitin proteasome is an essential component in the process of protein degradation and is intimately connected to cancer, we have made the decision to investigate the part that all of the known UbRGs play in LUAD. In this study, we conducted an analysis of the TCGA and GEO datasets to choose various UbRGs that were distinctly related to the outcomes of LUAD patients. We finished this work by employing a number of different statistical approaches. Based on the above results, we developed a novel and reliable risk model for the prediction of the outcomes of LUAD patients based on the screened UbRGs.

## 2. Materials and Methods

### 2.1. Ubiquitination-Related Genes and Gene Expression Data Collection

In this study, according to the relevance score greater than 5, 759 ubiquitination-related genes (UbRGs) were obtained from GeneCards database (https://www.genecards.org/).

The mRNA expression data and clinical characteristics of LUAD patients were obtained from TCGA and GEO. The TCGA-LUAD cohort included 59 normal and 535 LUAD patients, which were used for gene differential expression analysis. Among 535 LUAD patients, 49 patients were removed for unknown survival or survival time less than 30 days, and the remaining 486 patients were used for subsequent analyses. Two GEO cohorts, including GSE30219 (83 patients) and GSE31210 (226 patients), were used as validation cohorts. Additionally, gene methylation data were downloaded from TCGA.

### 2.2. Gene Differential Expression and Functional Enrichment Analyses

Differential UbRGs were screened using “limma” R package (logFC filter = 1and FDR filter = 0.05). Then, GO and KEGG analyses were conducted in Metascape website (https://metascape.org) [19]. The interaction between proteins was explored in STRING website (https://string-db.org).

### 2.3. Ubiquitination-Related Genes Signature Construction

To construct an UbRG signature for survival prediction, univariate Cox regression analysis was performed to identify significant prognostic UbRGs. Then, a risk-score signature was developed using the least absolute shrinkage and selection operator (LASSO) analysis, which can minimize the risk of overfitting. The expression value of LASSO-selected genes and correlation coefficients were used: risk-score = e^sum(each gene^'^s expression × corresponding coefficient)^. According to the median of risk-score, high-risk (HR) and low-risk (LR) groups were established. Survival (Kaplan–Meier method) analysis and ROC analysis were performed to test the prediction performance and stability of the signature. Univariate and multivariate assays were carried out to examine the independence of the signature.

### 2.4. Immune Landscapes Related to the Signature

To calculate the proportion of 22 tumor infiltrating immune cells, the CIBERSORT algorithm, which calculates the proportion of cells in the sample based on the expression of cell biomarkers [20], was performed. We also investigated the relationship between signature genes and immune cells. The score of immune-related functions in each sample was calculated using a single sample gene set enrichment analysis (ssGSEA).

### 2.5. Immune Gene Expression and Immunotherapy Response Prediction

The application of immune checkpoint inhibitor (ICI) has achieved great success in tumor treatment. At present, it mainly includes PD-1 and its ligands and CTLA4 and its ligands. In this study, we compared the expression of PD-1, PD-L1, PD-L2, and CTLA4 between the two risk groups to predict the difference in response to ICI between the two groups. Antigen presentation is a key step in initiating immune response. We explored the difference of the expression level of 21 HLA genes between the two groups.

The response to anti-PD-1 and anti-CTLA-4 can be estimated with the TIDE (tumor immune dysfunction and exclusion) algorithm [21]. The TIDE score was more accurate than the PD-L1 expression level in predicting the response to ICI drugs [22]. The higher the TIDE score, the worse the effect of antitumor immunity. We obtained the TIDE score of LUAD from the website (https://tide.dfci.harvard.edu).

### 2.6. Clinical Characteristics Subgroup Analysis

Subgroups were established according to the age, gender, and stage (≤65 and >65, female and male, stage I-II and stage III-IV), respectively. The survival analysis was conducted to test the applicability of the signature in different clinical characteristics subgroups.

### 2.7. Mutation Landscapes Related to the Signature

Tumor mutation burden (TMB) can also predict the effect of ICI therapy. The mutation landscape of the two groups was compared. The mutation landscapes of the two groups and signature genes were visualized using “maftools” package.

### 2.8. Gene Set Variation Analysis (GSVA)

We carried out gene set variation analysis (GSVA) using “GSVA” R packages to examine the variation in biological process activities in the studied samples based on the RNA-seq data in TCGA [23]. The study samples were divided into high- and low-risk subgroups and the comparison between them was performed in the signature scores of the gene sets.

### 2.9. Targeted Therapy Drug Prediction

We also obtained drug sensitivity information from CellMiner database (https://discover.nci.nih.gov/cellminer/). Then, we selected targeted therapy drugs for signature genes.

### 2.10. Statistical Methods

All statistical analyses were conducted using R software (version 4.1.3). Gene differential expression analysis, immune gene expression analysis, and the comparison of ssGSEA scores were performed using the Mann–Whitney test. The relationship between the targeted drug and signature genes was explored using the Pearson correlation test. The log-rank test and K–M analysis were applied to compare the OS between groups. *P* < 0.05 was considered statistically significant.

## 3. Results

### 3.1. Gene Differential Expression and Functional Enrichment Analyses

**Figure 1:**
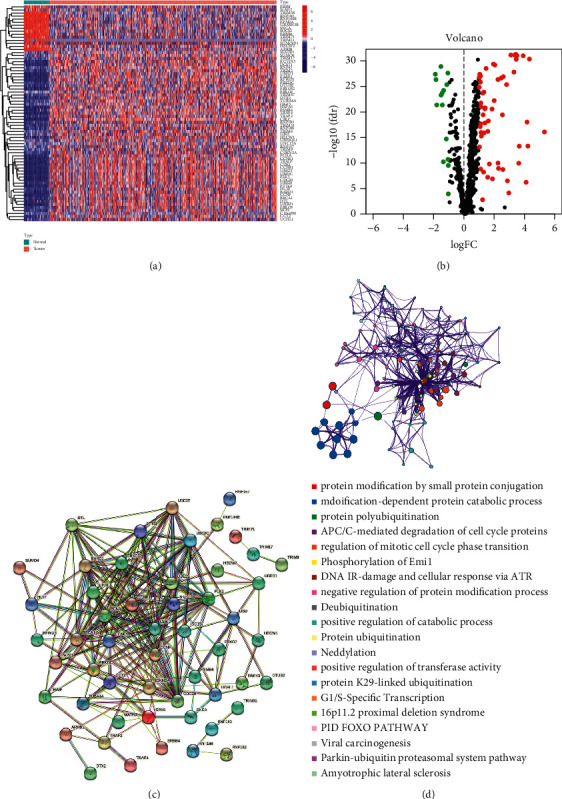
Visualization of differentially expressed UbRGs and functional enrichment analysis. (a) Heatmap. (b) Volcano. (c) Gene interaction prediction. (d) Functional enrichment analysis.

Total 71 UbRGs were differentially expressed, and 50 of these genes were visualized in heatmap (Figures 1(a) and 1(b)). The correlation between genes was found (Figure 1(c)). Functional enrichment analysis showed that these 71 UbRGs were largely related to cell cycle, protein modification, and catabolic process (Figure 1(d)). Our findings suggested that 71 UbRGs may be involved in tumor progression.

### 3.2. Ubiquitination-Related Genes Signature Construction

**Figure 2:**
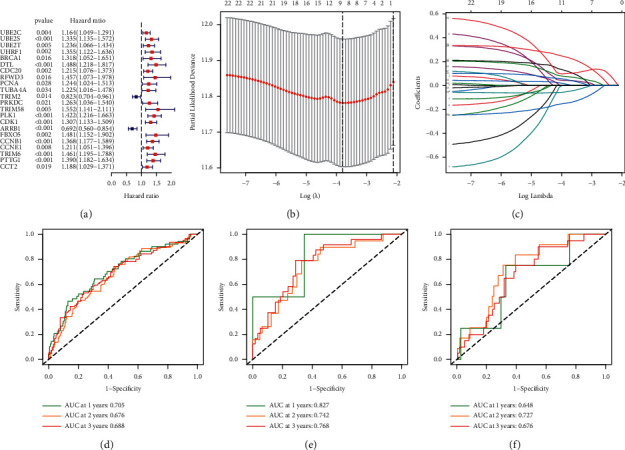
Construction and verification of the ubiquitination-related genes signature. (a) Prognosis-related UbRGs. (b) and (c) LASSO analysis. (d-f) ROC curves of data cohorts: (d) TCGA-LUAD. (e) GSE30219. (f) GSE31210.

**Figure 3:**
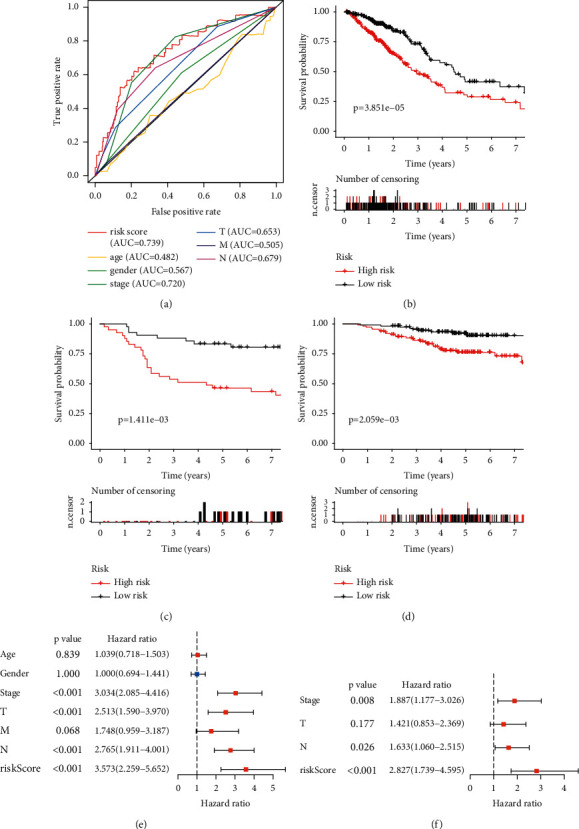
Testing the performance of the signature. (a) Multiple ROCs. (b-d) Survival analysis: (b) TCGA-LUAD. (c) GSE30219. (d) GSE31210. (e) Univariate Cox regression analysis. (f) Multivariate Cox regression analysis.

There were 22 genes associated with prognosis, including 2 protective genes and 20 risk genes (Figure 2(a)). The signature was: risk-score = (0.009846853 × TUBA4A) + (−0.049465962 × TRIM2) + (0.119625281 × TRIM58) + (0.101087198 × PLK1) + (−0.144720934 × ARRB1) + (0.043147457 × CCNB1) + (0.235201367 × TRIM6) + (0.074733269 × PTTG1) + (0.048466834 × CCT2) (Figures 2(b) and 2(c)). The AUCs of the TCGA-LUAD cohort at 1, 2, and 3 years were 0.705, 0.676, and 0.688, respectively (Figure 2(d)). The AUCs of two validation cohorts at 1, 2, and 3 year were 0.827, 0.742, and 0.768 (GSE30219) and 0.648, 0.727, and 0.676 (GSE31210) (Figures 2(e) and 2(f)), respectively. Compared with several available clinical characteristics, the AUC of the signature was the highest, which was 0.739 (Figure 3(a)). Survival analysis of all data cohorts revealed that the survival probability of the LR group was higher than that of the HR group (Figures 3(b)–3(d)). Univariate and multivariate Cox regression analysis results indicated that the signature could independently predict the prognosis of LUAD patients (Figures 3(e) and 3(f)).

### 3.3. Immune Landscapes Related to the Signature

**Figure 4:**
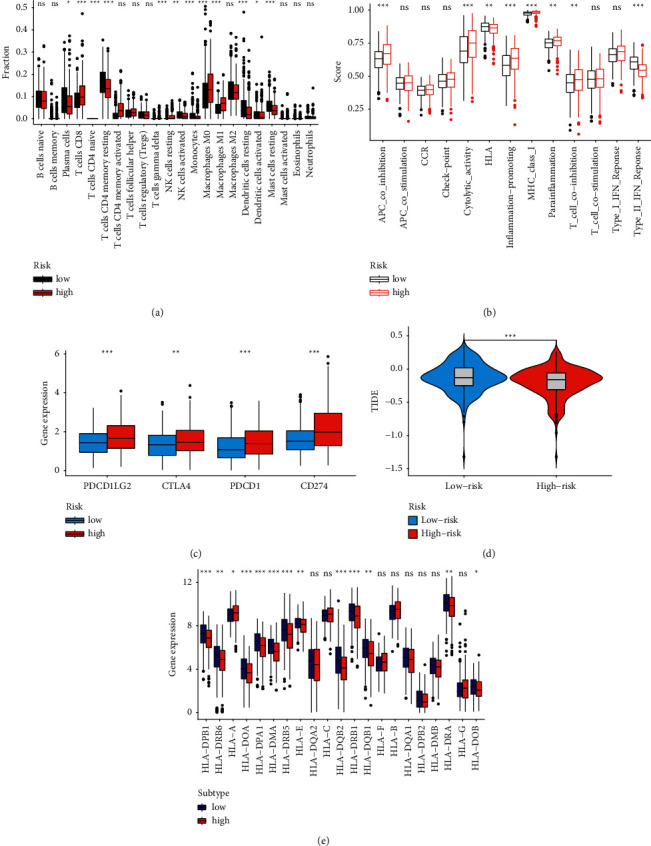
Immune landscapes related to the signature. (a) Immune cell infiltration analysis. (b) Immune-related function analysis. (c) Expression level of four immune checkpoints in the two risk groups. (d) TIDE scores of the two groups. (e) Expression level of HLA genes.

In the two risk groups, the abundances of CD8 T cells, activated CD4 T memory cells, resting NK cells, and macrophages were higher in the HR group, and the abundances of plasma cells, activated NK cells, and monocytes were higher in the LR group (Figure 4(a)).

Compared with the low expression group, the infiltration of CD8 T cells was higher in the high CCNB1/CCT2/PLK1/PTTG1 expression groups, the infiltration of resting CD4 T memory cells was higher in the high ARRB1/CCNB1/TRIM2 expression groups, and the infiltration of activated CD4 T memory cells was higher in the high CCT2/TRIM6/TUBA4A expression groups. Additionally, the infiltration of monocytes was higher in the high ARRB1/TRIM2/TRIM58 expression groups (Figure S1).

The ssGSEA results demonstrated that there were differences between the two risk groups in APC co-inhibition, T cell co-inhibition, inflammation promoting, and so on (Figure 4(b)).

### 3.4. Immune Gene Expression and Immunotherapy Response Prediction

The expressions of PD-1, PD-L1, PD-L2, and CTLA4 were all higher in the HR group (Figure 4(c)). The TIDE score of the HR group was lower than that in the LR group (Figure 4(d)). The expressions of HLA-D and HLA-A were higher in the HR group (Figure 4(e)). These results indicated that the HR group responded better to ICI therapy than the LR group.

### 3.5. Mutation Landscapes Related to the Signature

**Figure 5:**
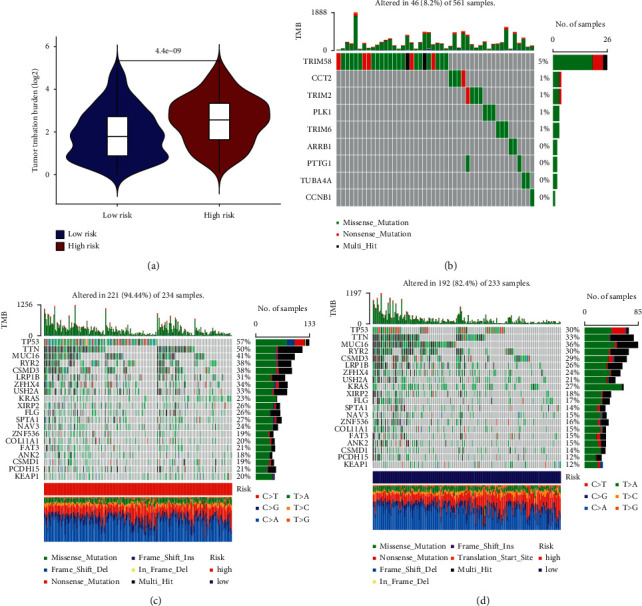
Tumor mutation burden analysis. (a) Comparison of TMB between the two groups. (b) Mutation analysis of signature genes. (c) TMB of the HR group. (d) TMB of the LR group.

The TMB of the HR group was statistically higher than that of the LR group, indicating that the HR group responded better to ICI therapy than the LR group, which was consisted with the results of immune analysis (Figure 5(a)). The mutation analysis of signature genes showed that there were mutations in TRIM6, TRIM58, CCT2, TRIM2, and PLK1, while there was no mutation in CCNB1, TUBA4A, PTTG1, and ARRB1(Figure 5(b)). The specific gene mutations of the two risk groups were significantly different (Figures 5(c) and 5(d)). The mutation rate of TP53 was high as 57%, while it was 30% in the LR group. The Missense mutation was the highest mutation type in both groups. The data of SNP revealed that the most common type of the two risk groups was *C* > *A*.

### 3.6. Clinical Characteristics Subgroup Analysis

**Figure 6:**
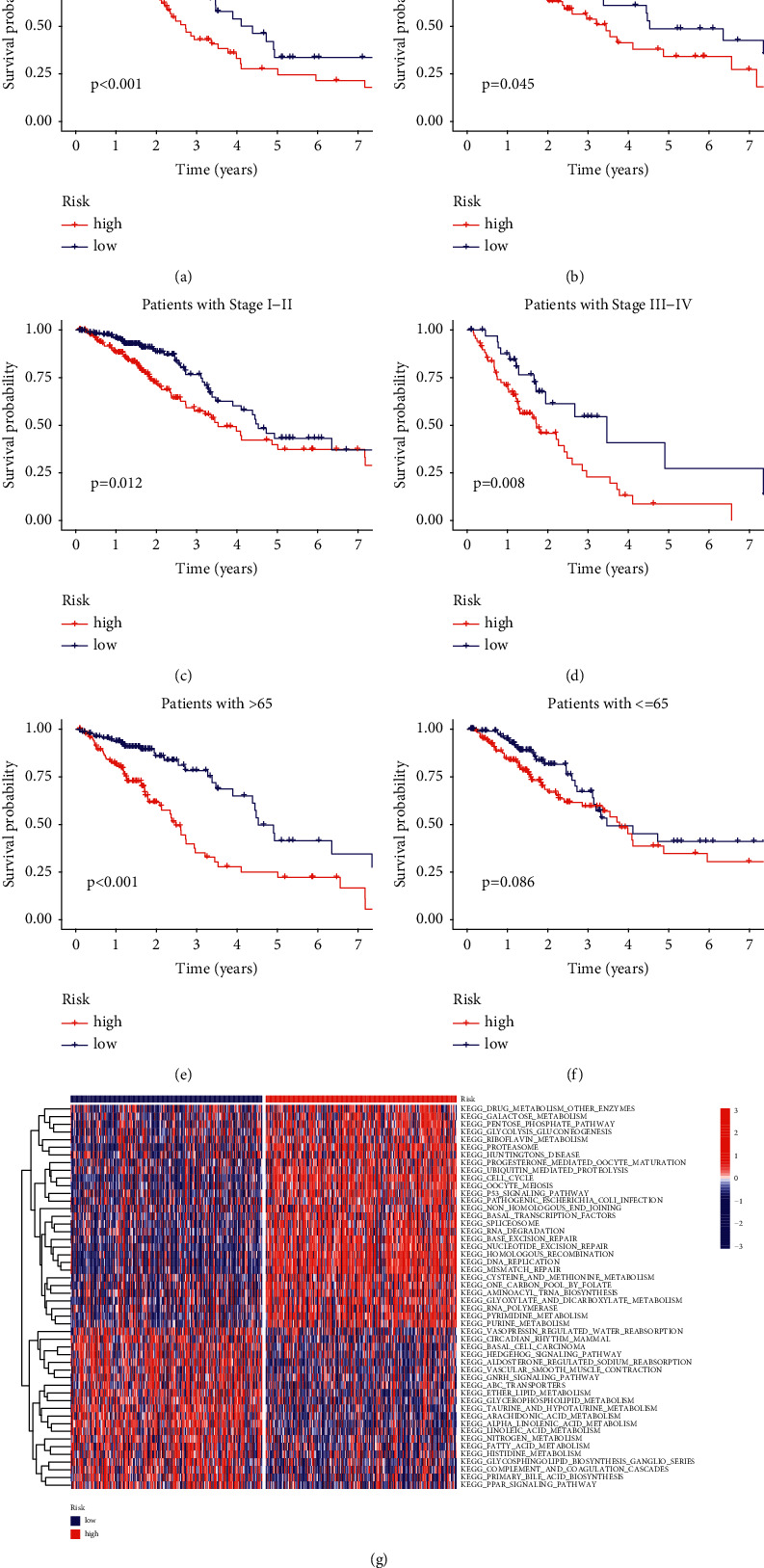
Clinical characteristics subgroup analysis and GSVA. (a) Female group. (b) Male group. (c) Stage I−II group. (d) Stage III−IV group. (e) >65 group. (f) ≤65 group. (g) GSVA.

It has been found that the signature was suitable for different clinical groups. In the subgroups of gender and stage, the survival probability of the HR group was lower than that than of the LR group (Figures 6(a)–6(d)). In the subgroups of age, the survival analysis results of the >65 group were statistically different, but there was no difference in the ≤65 group (Figures 6(e) and 6(f)).

### 3.7. Gene Set Variation Analysis

The results of GSVA showed that multiple metabolic-related pathways (cysteine and methionine metabolism, pyrimidine metabolism, and so on), P53 signal pathway and RNA degradation, were active in the HR group. Other multiple metabolic-related pathways (fatty acid metabolism, nitrogen metabolism and so on), PPAR signaling pathway and GNRH signaling pathway, were active in the LR group (Figure 6(g)).

### 3.8. Targeted Therapy Drug Prediction

**Figure 7:**
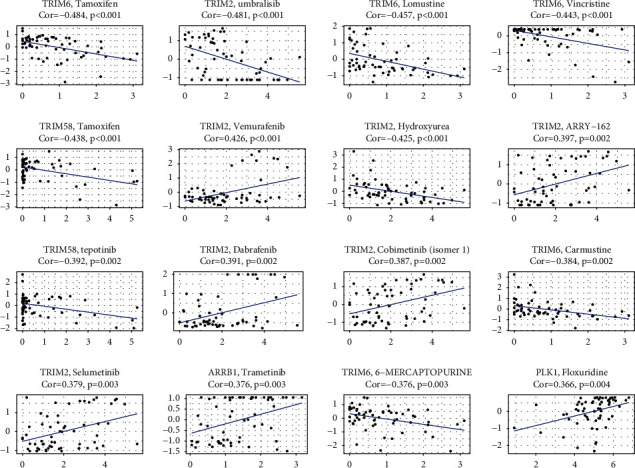
Targeted therapy drugs prediction.

Multiple drugs were screened out and the first 16 results were visualized (Figure 7). It showed that CCNB1, CCT2, PLK1, and PTTG1 were all sensitive to 6-thioguanine. ARRB1 and TRIM2 were sensitive to dabrafenib, while TUBA4A was resistant to it. TRIM6 was sensitive to gefitinib. Apart from cladribine and fludarabine, TRIM58 was resistant to many drugs. The specific results are provided in the supplementary table (Table S1). These analyses above can provide reference for the selection of clinical therapeutic drugs.

## 4. Discussion

With a survival rate of fewer than 18% after five years, LUAD, which is characterized by a tendency toward an advanced stage and metastatic tumor, has worse survival outcomes than many other forms of cancer [24, 25]. Patients diagnosed with LUAD at an early stage can, in theory, better survive from the disease; however, this category only accounts for 25–30% of LUAD cases [26, 27]. In the early stages of LUAD, surgical resection with or without further adjuvant chemotherapy is considered as the cornerstone of therapeutic treatment, and the TNM stage is the most useful marker to predict outcomes traditionally [28]. In contrast, survival rates are highly variable, even among LUAD patients in the same stage who have received the same treatment; this highlights the genetic diversity of patients with LUAD.

In recent years, a large number of cancer-related research studies have focused on a few functional gene profiles that have been found to have a significant impact on the development and progression of cancer [29, 30]. Importantly, several studies have reported the important roles of ubiquitination-related genes in the development of various tumors including LUAD. For instance, the levels of UBE2C in tissues with NSCLC were found to be considerably greater than those in equivalent normal tissues. An increased level of the UBE2C expression is linked to angiogenesis and a bad prognosis. In addition, aberrant activation of UBE2C enhanced cell proliferation, clonogenicity, and invasive growth of NSCLC [31]. Jia et al. reported that RFWD3 was overexpressed in gastric carcinoma, and its knockdown inhibited the proliferation and migration of gastric carcinoma cells through modulating AKT, ERK/P38, and Slug pathways [32]. In the current research study, we used the mRNA expression data of 71 UbRGs from the TCGA datasets and the LASSO regression analysis to construct a 9-UbRGs predictive signature. A functional study demonstrated that the differentially expressed UbRGs were closely associated with cancer and revealed that the dysregulation of ubiquitination is critical in the initiation and progression of cancer. Importantly, the high-risk patients with LUAD had shorter OS than the low-risk patients. The predictive value of the signature was validated by employing two different internal validation cohorts (GSE30219 and GSE31210 datasets) because there was a risk that the signature was overtrained while it was being constructed. The results suggested that the novel signature was robust and reproducible in patients with LUAD. Because the area under the curve (AUC) in both the TCGA dataset and the validation dataset was more than 0.7, it was clear that the new signature had a degree of success in predicting survival. The novel signature showed promise as a potential independent prognostic factor, according to the results of both univariate and multivariate analyses, which were used to evaluate the TCGA dataset of patients diagnosed with LUAD.

The LUAD TME status can be determined by analyzing the relationship between immune cell infiltration and the LUAD gene signature, which was previously thought to be a useful indicator for predicting disease outcomes and immunotherapy response in malignancies [33, 34]. In this study, by using the CIBERSORT algorithm, we were able to determine the relative abundance of 22 distinct types of immune cells that were present in the LUAD sample. According to the findings of our study, the high-risk group had a tendency to have a lower immune cell infiltration rate, which suggests that the activation of immune cells may have a positive correlation with a better prognosis. We observed that the abundance of CD8 T cells, activated CD4 T memory cells, resting NK cells, and macrophages was higher in the HR group. The ssGSEA results demonstrated that there were differences between the two risk groups in APC co-inhibition, T cell co-inhibition, inflammation promoting, and so on. In the therapeutic treatment of a variety of malignancies, ICIs have demonstrated efficacy that is encouraging [35, 36]. Cancer patients who are being treated with ICIs would benefit from the identification of predictive biomarkers due to the fact that different individuals demonstrated varying responses to ICIs. Although PD-L1 has been suggested as a marker that was favorably related to the efficacy of ICI, the use of PD-L1 alone is not sufficient for diagnosing cancer in patients. Jiang et al. created the TIDE algorithm to predict the responses to ICIs by characterizing dysfunctional T cells and infiltrating the cytotoxic T lymphocytes (CTLs) level [21]. This was done in order to determine how well the algorithm would work. In our study, we discovered that immunological checkpoints had a significantly higher level of expression in the high-risk group when stratified by a novel signature. Patients with similar expression levels of immunological checkpoints might be distinguished from one another using the novel signature. The fact that a low-risk group's TIDE score was much lower than that of the high-risk group confirmed our hypothesis, which stated that a high TIDE score suggested a suboptimal response to ICI treatment. As a result, the signature of the UbRG might make it easier to implement ICI for the treatment of LUAD.

The identification of the TMB is now a regular experiment, thanks to the advancement of technology that allows for DNA sequencing [37]. Several clinical investigations have shown that the TMB can be used as a predictor of how well ICIs would work as treatments [38, 39]. This role of the TMB as a biomarker has been established. In recent years, TMB has been demonstrated to be an important marker for predicting response to immune checkpoint inhibitors in several types of tumors, such as cervical cancer, rectal cancer, and bladder cancer [40–42]. In addition, there are a growing number of researchers who are beginning to investigate the connection between TMB and LUAD [43, 44]. TMB was found to have a close relationship with immune-mediated survival in LUAD. We observed that the TMB of the HR group was statistically higher than that of the LR group, indicating that the HR group responded better to ICI therapy than the LR group, which is consistent with the results of immune analysis.

Increased expressions of several genes related the long-term survivals were related to increased drug resistance for a number of chemotherapy drugs that are approved by the FDA, such as decitabine, homoharringtonine, pipobroman, lxazomib citrate, and tamoxifen [45, 46]. These findings were based on the analysis of data from NCI-60 cell lines. Hence, we also observed some prognostic genes that were related to increased drug sensitivity of a few drugs. It showed that CCNB1, CCT2, PLK1, and PTTG1 were all sensitive to 6-thioguanine. ARRB1 and TRIM2 were sensitive to dabrafenib, while TUBA4A was resistant to it. TRIM6 was sensitive to gefitinib. Apart from cladribine and fludarabine, TRIM58 was resistant to many drugs. These analyses above can provide reference for the selection of clinical therapeutic drugs.

However, our research has some limitations that must be taken into account. First, our research relied heavily on data obtained from TCGA, in which the majority of patients were either White or Asian. The application of our findings to patients of other ethnicities should be approached with extreme caution. Second, in vitro and in vivo experiments were needed to further confirm our findings.

## 5. Conclusion

Our study identified a prognostic signature based on nine UbRGs to predict the overall survivals of LUAD patients. It was confirmed that there was a close association between the risk scores and the progression and immune infiltration of LUAD. Because it is possible to estimate the IC50 of chemotherapeutic medicines using the signature, the signature may have some clinical importance. The prognostic signature has the potential to accurately predict the outcome in LUAD and may make it easier to establish tailored therapy programs for the immune system.

## Data Availability

The datasets used and/or analysed during the current study are available from the corresponding author on reasonable request.

## Supplementary Materials

Supplementary Materials Figure S1 Analysis on the association between 9 signature genes and 22 immune cells. (A) ARRB1. (B) CCNB1. (C) CCT2. (D) PLK1. (E) PTTG1. (F) TRIM2. (G) TRIM58. (H) TRIM6. (I) TUBA4A. Table S1. All results of targeted therapy drugs prediction.
