# Supplementary material for: Prognostic Signature, Immune Features, and Therapeutic Responses of a Novel Ubiquitination-Related Gene Signature in Lung Adenocarcinoma
Source: J Oncol. 2022 Aug 16;2022:2524649. doi: 10.1155/2022/2524649 (PMC9398812; doi:10.1155/2022/2524649)
Supplement: Supplementary Materials — Figure S1 Analysis on the association between 9 signature genes and 22 immune cells. (A) ARRB1. (B) CCNB1. (C) CCT2. (D) PLK1. (E) PTTG1. (F) TRIM2. (G) TRIM58. (H) TRIM6. (I) TUBA4A. Table S1. All results of targeted therapy drugs prediction. [file 2524649.f1.zip › Table S1.docx]

Table S1. All results of targeted therapy drugs prediction.

| Gene | Drug | cor | pvalue |
| --- | --- | --- | --- |
| ARRB1 | Trametinib | 0.376428 | 0.003033 |
| ARRB1 | Cobimetinib (isomer 1) | 0.351023 | 0.005961 |
| ARRB1 | Ifosfamide | -0.34904 | 0.00627 |
| ARRB1 | ARRY-162 | 0.341752 | 0.007529 |
| ARRB1 | Selumetinib | 0.341471 | 0.007582 |
| ARRB1 | Encorafenib | 0.337579 | 0.008345 |
| ARRB1 | Dabrafenib | 0.318316 | 0.013188 |
| ARRB1 | MITOXANTRONE | -0.31613 | 0.013866 |
| ARRB1 | Vemurafenib | 0.305126 | 0.017759 |
| ARRB1 | Mitoxantrone | -0.3028 | 0.01869 |
| ARRB1 | Midostaurin | -0.30268 | 0.018739 |
| ARRB1 | BMN-673 | -0.27067 | 0.036463 |
| ARRB1 | Temsirolimus | -0.27052 | 0.036571 |
| ARRB1 | Fulvestrant | 0.26065 | 0.04428 |
| CCNB1 | Denileukin Diftitox Ontak | -0.35347 | 0.005598 |
| CCNB1 | 6-Thioguanine | 0.340144 | 0.007835 |
| CCNB1 | Allopurinol | 0.268667 | 0.037927 |
| CCT2 | Nelarabine | 0.305546 | 0.017594 |
| CCT2 | 6-Thioguanine | 0.301232 | 0.019343 |
| CCT2 | Allopurinol | 0.294528 | 0.022352 |
| CCT2 | Vinorelbine | -0.28127 | 0.02948 |
| CCT2 | Ifosfamide | 0.277942 | 0.031539 |
| CCT2 | Vorinostat | 0.273598 | 0.034409 |
| CCT2 | Hydroxyurea | 0.272645 | 0.035067 |
| CCT2 | Paclitaxel | -0.25953 | 0.04523 |
| PLK1 | Floxuridine | 0.365603 | 0.004072 |
| PLK1 | Gemcitabine | 0.350346 | 0.006065 |
| PLK1 | Pemetrexed | 0.349798 | 0.00615 |
| PLK1 | Acrichine | 0.317769 | 0.013355 |
| PLK1 | 6-Thioguanine | 0.315362 | 0.014113 |
| PLK1 | 6-THIOGUANINE | 0.30972 | 0.016033 |
| PLK1 | Thiotepa | 0.295303 | 0.021985 |
| PLK1 | Triethylenemelamine | 0.291913 | 0.023628 |
| PLK1 | Raltitrexed | 0.290212 | 0.024491 |
| PLK1 | Cladribine | 0.286856 | 0.026272 |
| PLK1 | Cytarabine | 0.285812 | 0.026848 |
| PLK1 | tfdu | 0.281556 | 0.029306 |
| PLK1 | 6-MERCAPTOPURINE | 0.271672 | 0.03575 |
| PLK1 | Cisplatin | 0.268411 | 0.038118 |
| PLK1 | Vinorelbine | -0.26299 | 0.042338 |
| PLK1 | METHOTREXATE | 0.262079 | 0.043088 |
| PLK1 | Denileukin Diftitox Ontak | -0.26074 | 0.044202 |
| PLK1 | Chlorambucil | 0.25919 | 0.045526 |
| PLK1 | Fludarabine | 0.2578 | 0.046739 |
| PLK1 | Melphalan | 0.254336 | 0.049877 |
| PTTG1 | Denileukin Diftitox Ontak | -0.33823 | 0.008213 |
| PTTG1 | 6-Thioguanine | 0.324468 | 0.01143 |
| TRIM2 | umbralisib | -0.48068 | 0.000101 |
| TRIM2 | Vemurafenib | 0.426373 | 0.000682 |
| TRIM2 | Hydroxyurea | -0.42472 | 0.000719 |
| TRIM2 | ARRY-162 | 0.396784 | 0.001697 |
| TRIM2 | Dabrafenib | 0.391162 | 0.001999 |
| TRIM2 | Cobimetinib (isomer 1) | 0.387499 | 0.002222 |
| TRIM2 | Selumetinib | 0.378576 | 0.002858 |
| TRIM2 | DIGOXIN | -0.36238 | 0.004436 |
| TRIM2 | Dasatinib | -0.35749 | 0.005044 |
| TRIM2 | MITOXANTRONE | -0.35416 | 0.0055 |
| TRIM2 | Encorafenib | 0.353663 | 0.005571 |
| TRIM2 | Uracil mustard | -0.34217 | 0.007452 |
| TRIM2 | Temsirolimus | -0.3409 | 0.00769 |
| TRIM2 | Pemetrexed | -0.34067 | 0.007733 |
| TRIM2 | RAPAMYCIN | -0.3383 | 0.008198 |
| TRIM2 | Chlorambucil | -0.33563 | 0.008752 |
| TRIM2 | TYROTHRICIN | 0.327037 | 0.010757 |
| TRIM2 | Nitrogen mustard | -0.32626 | 0.010957 |
| TRIM2 | Melphalan | -0.32058 | 0.012516 |
| TRIM2 | Etoposide | -0.31761 | 0.013404 |
| TRIM2 | 6-THIOGUANINE | -0.31367 | 0.014667 |
| TRIM2 | Asparaginase | -0.3102 | 0.015862 |
| TRIM2 | Trametinib | 0.305833 | 0.017483 |
| TRIM2 | Triethylenemelamine | -0.30575 | 0.017515 |
| TRIM2 | Thiotepa | -0.29931 | 0.020167 |
| TRIM2 | Gemcitabine | -0.29822 | 0.020647 |
| TRIM2 | 6-Mercaptopurine | -0.29808 | 0.020711 |
| TRIM2 | Mitoxantrone | -0.29122 | 0.023976 |
| TRIM2 | Mitotane | -0.28971 | 0.02475 |
| TRIM2 | tfdu | -0.28809 | 0.025606 |
| TRIM2 | Valrubicin | -0.2805 | 0.029944 |
| TRIM2 | 6-Thioguanine | -0.2785 | 0.031187 |
| TRIM2 | Allopurinol | -0.27777 | 0.031651 |
| TRIM2 | Cladribine | -0.26752 | 0.038787 |
| TRIM2 | Everolimus | -0.26627 | 0.039745 |
| TRIM2 | Raltitrexed | -0.26449 | 0.041136 |
| TRIM2 | Cytarabine | -0.26352 | 0.041914 |
| TRIM2 | Fulvestrant | -0.25915 | 0.045558 |
| TRIM58 | Tamoxifen | -0.4379 | 0.000467 |
| TRIM58 | tepotinib | -0.39221 | 0.001939 |
| TRIM58 | Fludarabine | 0.357434 | 0.005052 |
| TRIM58 | Sulfatinib | -0.30913 | 0.016247 |
| TRIM58 | Copanlisib | -0.30785 | 0.016717 |
| TRIM58 | Mithramycin | -0.29563 | 0.02183 |
| TRIM58 | Actinomycin D | -0.29124 | 0.023964 |
| TRIM58 | Depsipeptide | -0.28665 | 0.026386 |
| TRIM58 | Bortezomib | -0.27698 | 0.032156 |
| TRIM58 | Palbociclib | -0.27673 | 0.032319 |
| TRIM58 | Carfilzomib | -0.27472 | 0.033649 |
| TRIM58 | Homoharringtonine | -0.26288 | 0.042431 |
| TRIM58 | LEE-011 | -0.25887 | 0.045806 |
| TRIM58 | Cladribine | 0.255578 | 0.048733 |
| TRIM58 | IPI-145 | -0.25552 | 0.048785 |
| TRIM6 | Tamoxifen | -0.48432 | 8.82E-05 |
| TRIM6 | Lomustine | -0.45676 | 0.000244 |
| TRIM6 | Vincristine | -0.44271 | 0.000397 |
| TRIM6 | Carmustine | -0.38406 | 0.00245 |
| TRIM6 | 6-MERCAPTOPURINE | -0.37601 | 0.003069 |
| TRIM6 | Ifosfamide | -0.35558 | 0.005301 |
| TRIM6 | Dasatinib | 0.354131 | 0.005504 |
| TRIM6 | Vandetanib | 0.349433 | 0.006208 |
| TRIM6 | Ixabepilone | -0.3479 | 0.006453 |
| TRIM6 | Irofulven | 0.345578 | 0.006843 |
| TRIM6 | Vinblastine | -0.34221 | 0.007443 |
| TRIM6 | Pipamperone | -0.33303 | 0.009322 |
| TRIM6 | VINORELBINE | -0.3274 | 0.010665 |
| TRIM6 | Erlotinib | 0.31489 | 0.014266 |
| TRIM6 | Paclitaxel | -0.30527 | 0.0177 |
| TRIM6 | Oxaliplatin | -0.30281 | 0.018688 |
| TRIM6 | DAUNORUBICIN | -0.30182 | 0.019095 |
| TRIM6 | Gefitinib | 0.299735 | 0.019983 |
| TRIM6 | Fluorouracil | -0.29844 | 0.020549 |
| TRIM6 | Acrichine | -0.29346 | 0.022865 |
| TRIM6 | Estramustine | -0.29135 | 0.023912 |
| TRIM6 | Homoharringtonine | -0.28751 | 0.025918 |
| TRIM6 | Eribulin mesilate | -0.28323 | 0.028317 |
| TRIM6 | Carfilzomib | -0.27951 | 0.030551 |
| TRIM6 | Simvastatin | 0.279221 | 0.030733 |
| TRIM6 | Sonidegib | 0.266985 | 0.039193 |
| TRIM6 | Crizotinib | -0.26477 | 0.040911 |
| TRIM6 | TYROTHRICIN | -0.26246 | 0.042778 |
| TUBA4A | Vemurafenib | -0.33126 | 0.009727 |
| TUBA4A | Dabrafenib | -0.32259 | 0.011943 |
| TUBA4A | Actinomycin D | -0.30793 | 0.016687 |
| TUBA4A | Epirubicin | -0.29439 | 0.022419 |
| TUBA4A | Mithramycin | -0.28564 | 0.026944 |
| TUBA4A | Afatinib | 0.283071 | 0.02841 |
| TUBA4A | Bortezomib | -0.27739 | 0.031892 |
| TUBA4A | Arsenic trioxide | -0.27632 | 0.032587 |
| TUBA4A | Vincristine | -0.2743 | 0.033934 |
| TUBA4A | Everolimus | 0.26621 | 0.039789 |
| TUBA4A | brigatinib | 0.263515 | 0.041917 |
| TUBA4A | Pipamperone | -0.26138 | 0.043666 |
