# Supplementary figures and images for: Prognostic Signature, Immune Features, and Therapeutic Responses of a Novel Ubiquitination-Related Gene Signature in Lung Adenocarcinoma
Source: J Oncol. 2022 Aug 16;2022:2524649. doi: 10.1155/2022/2524649 (PMC9398812; doi:10.1155/2022/2524649)

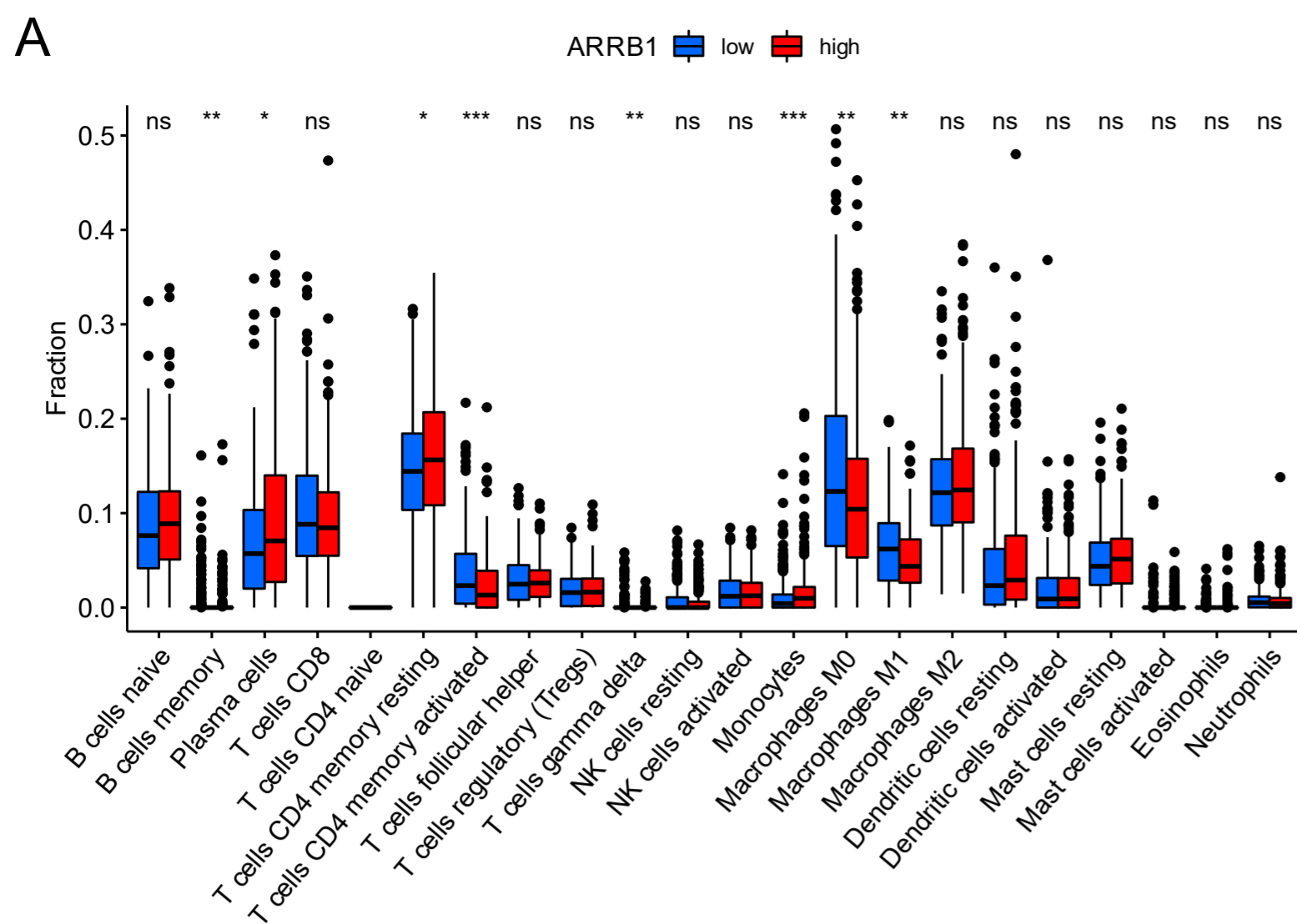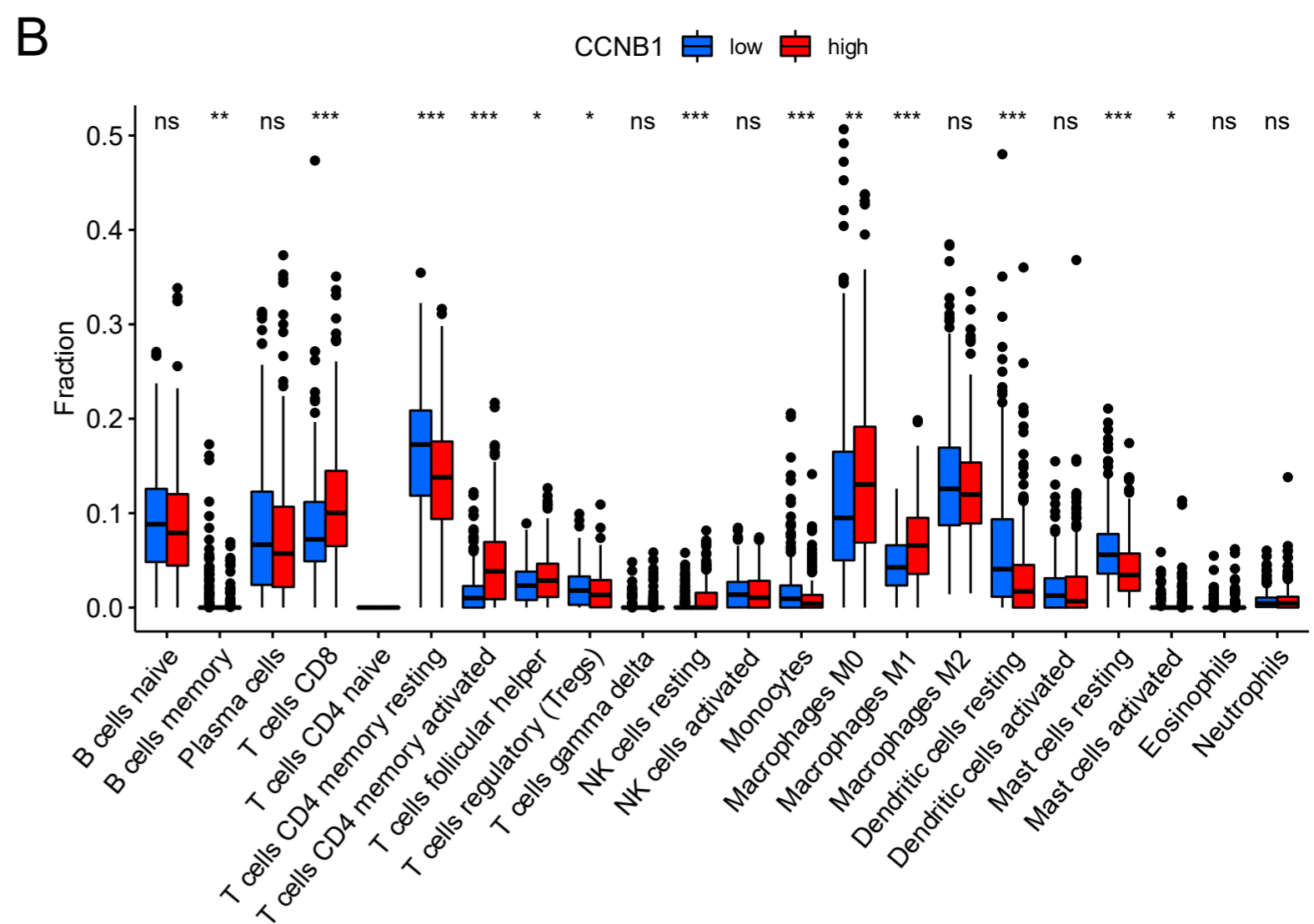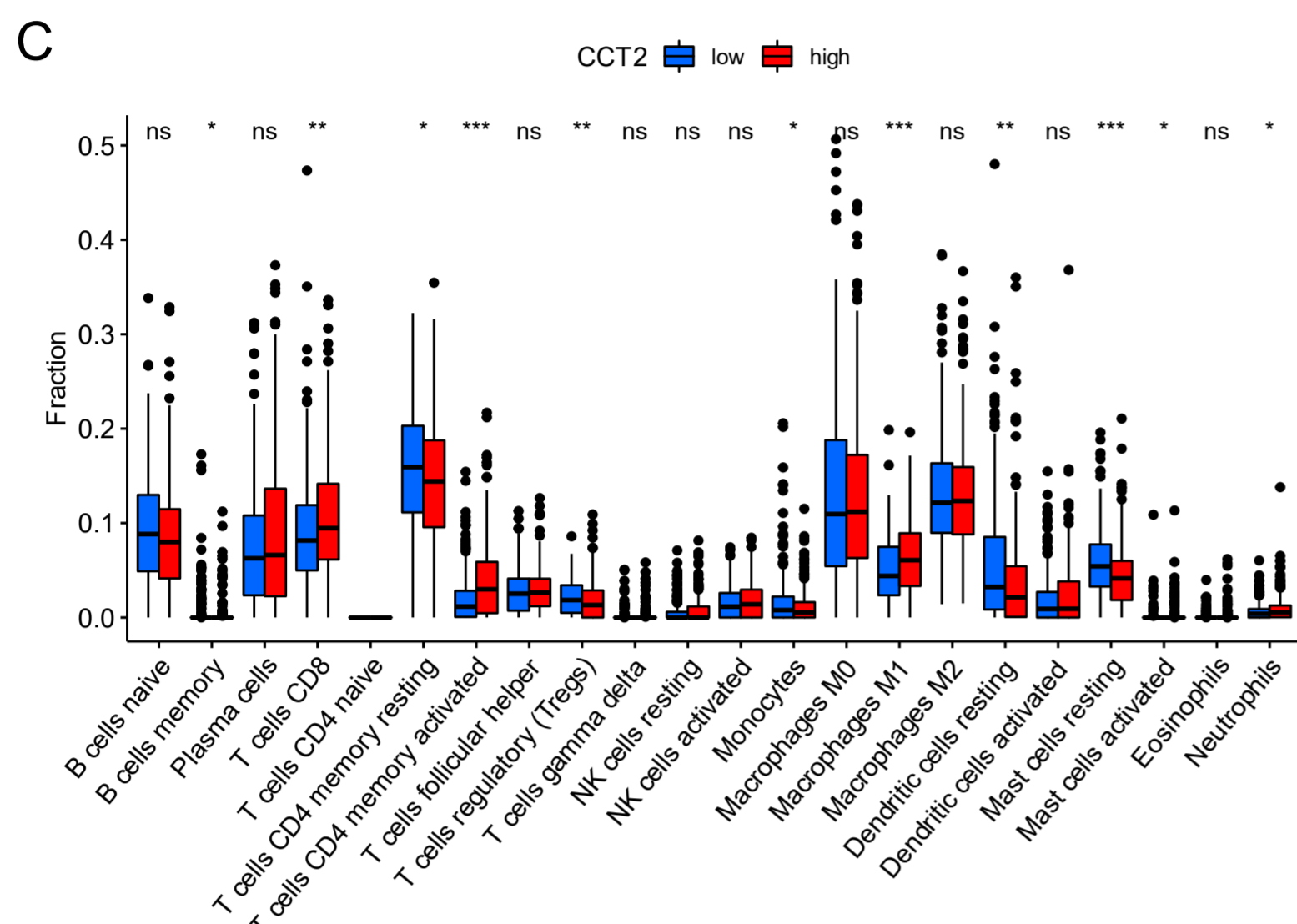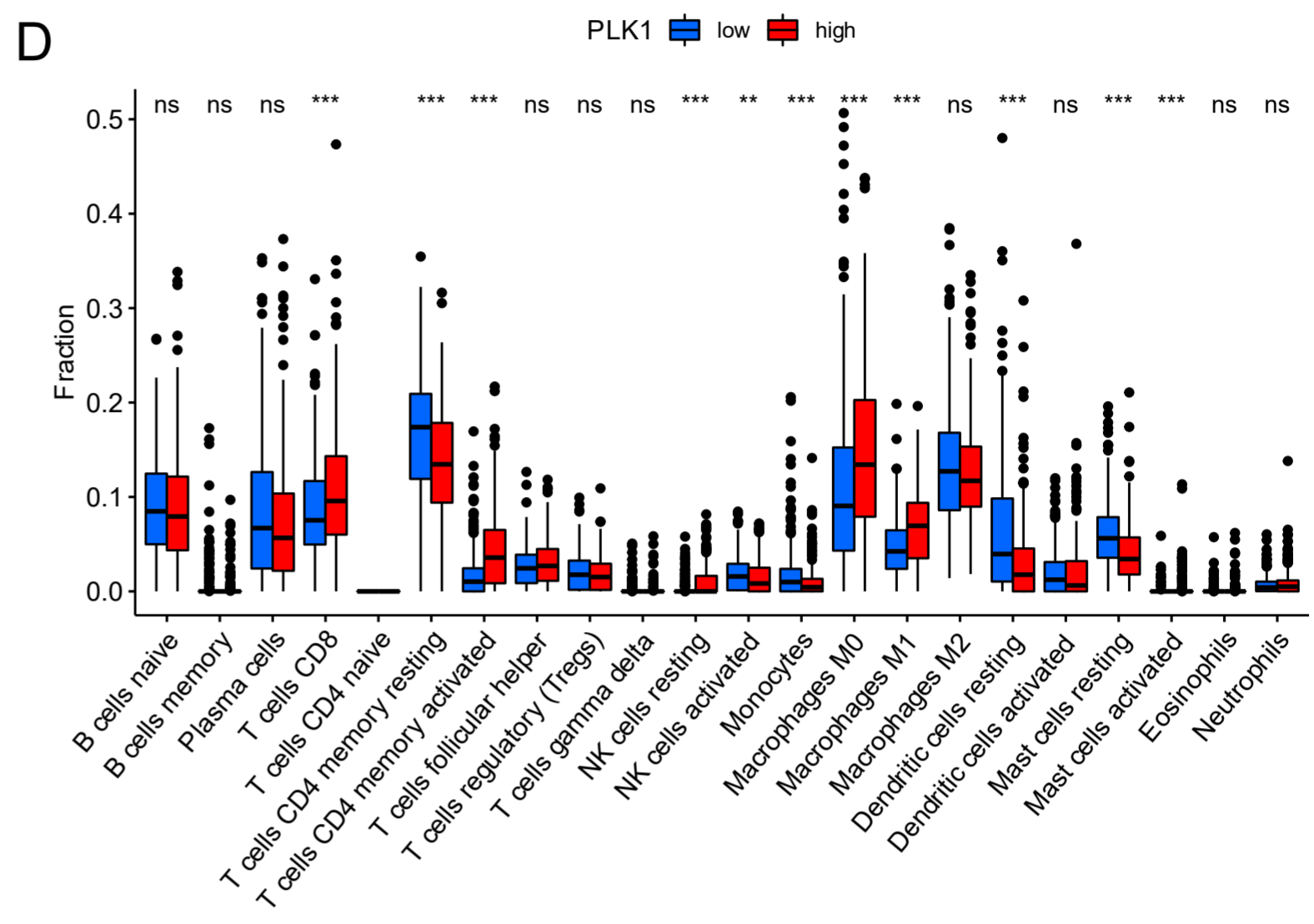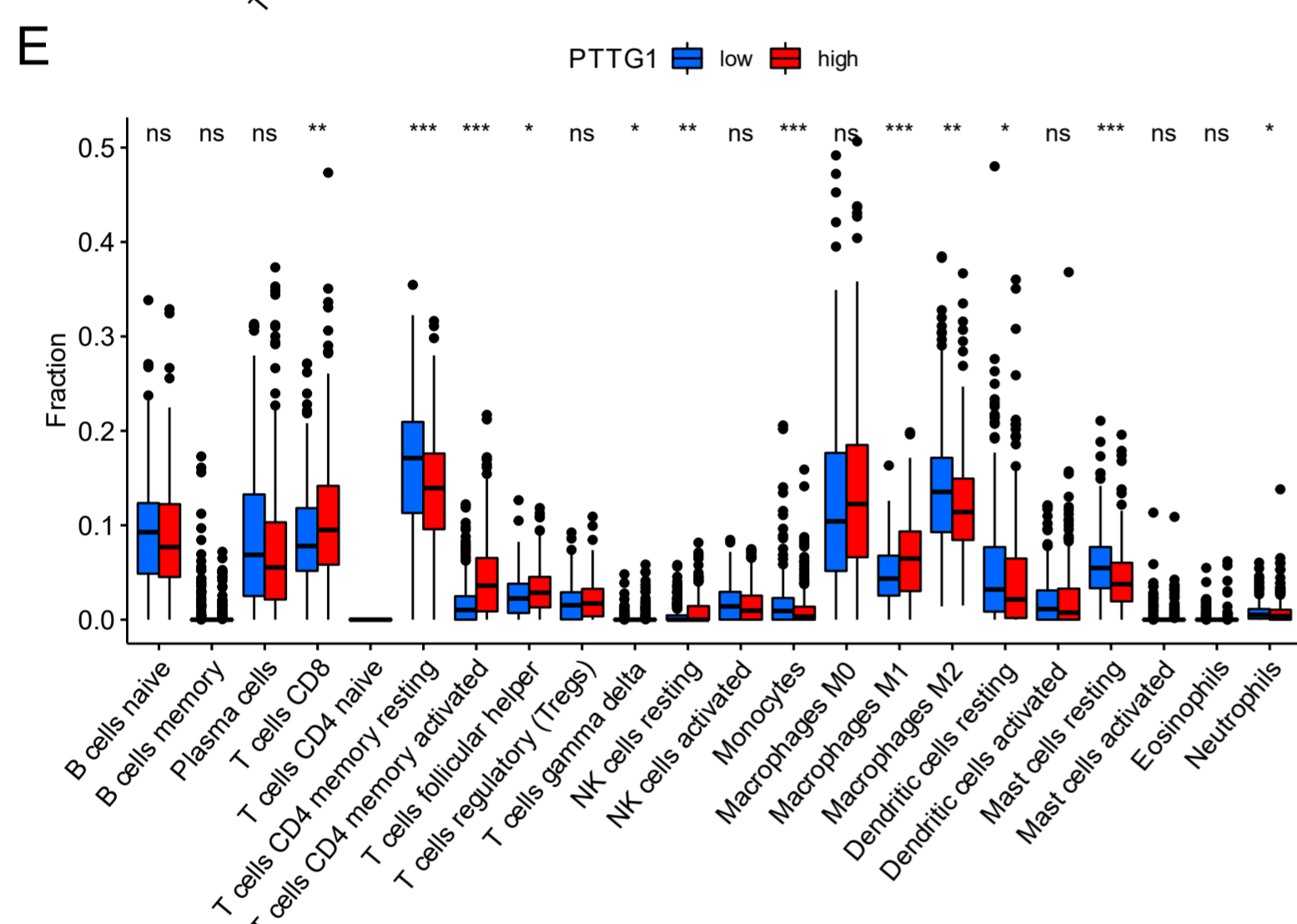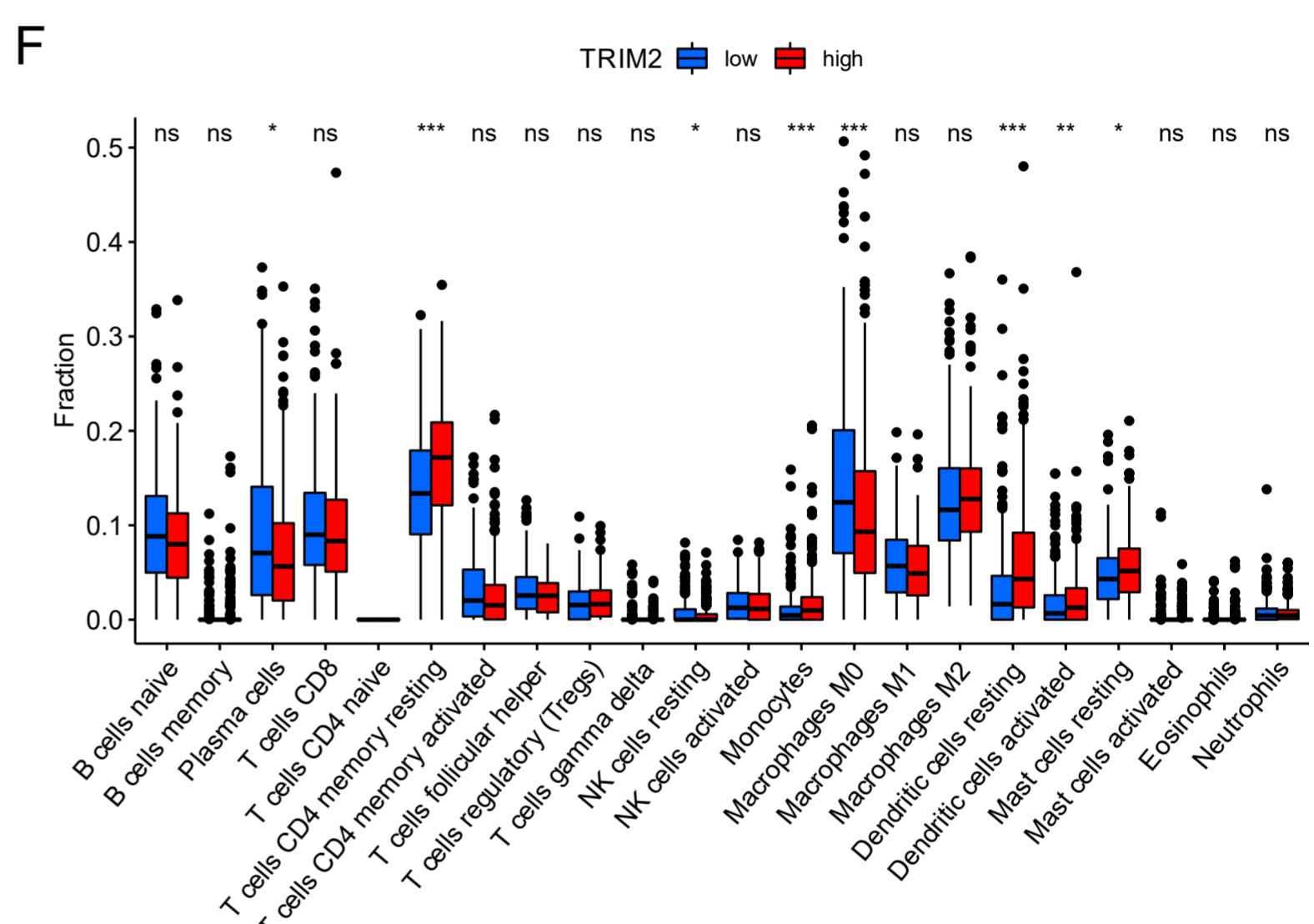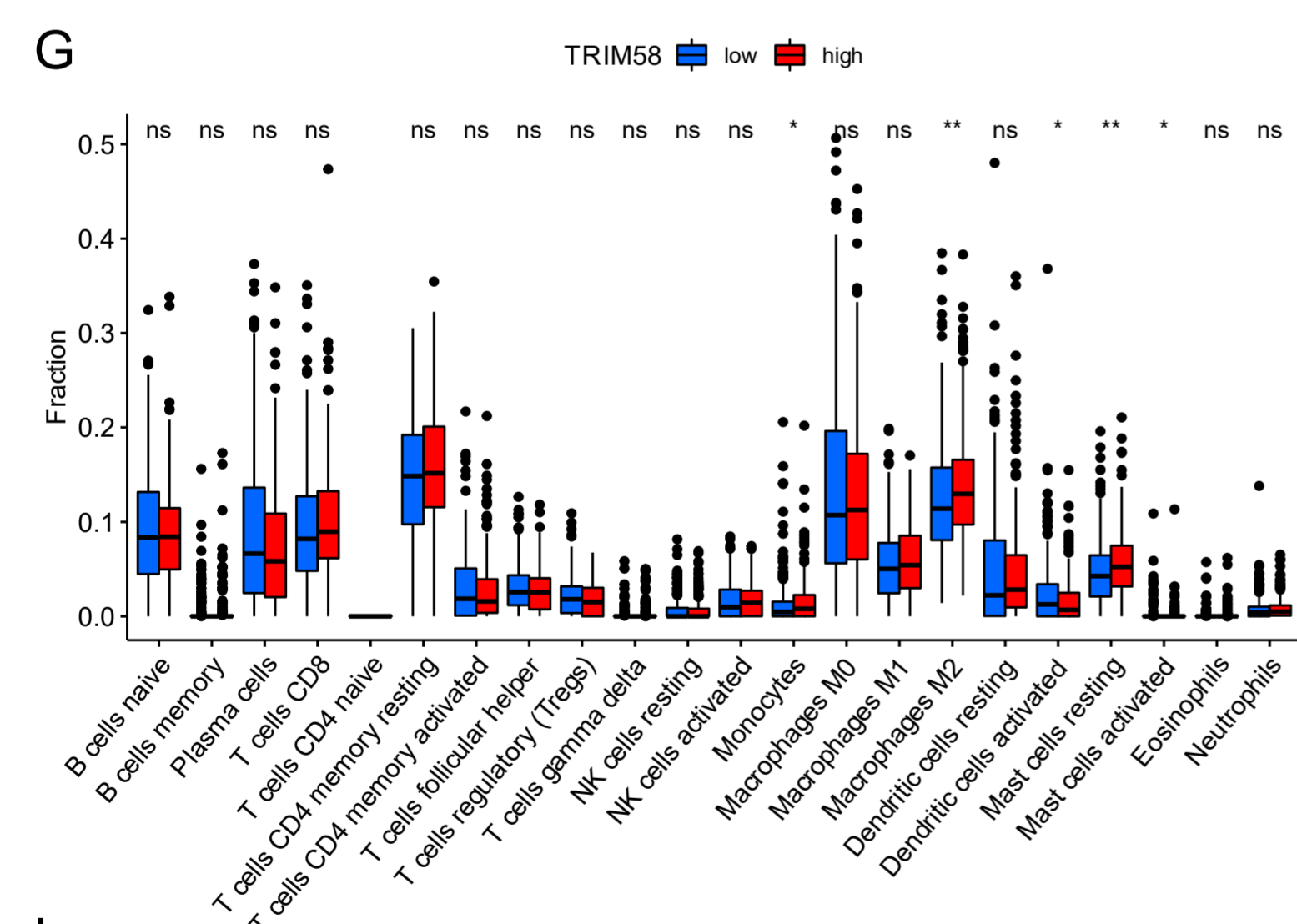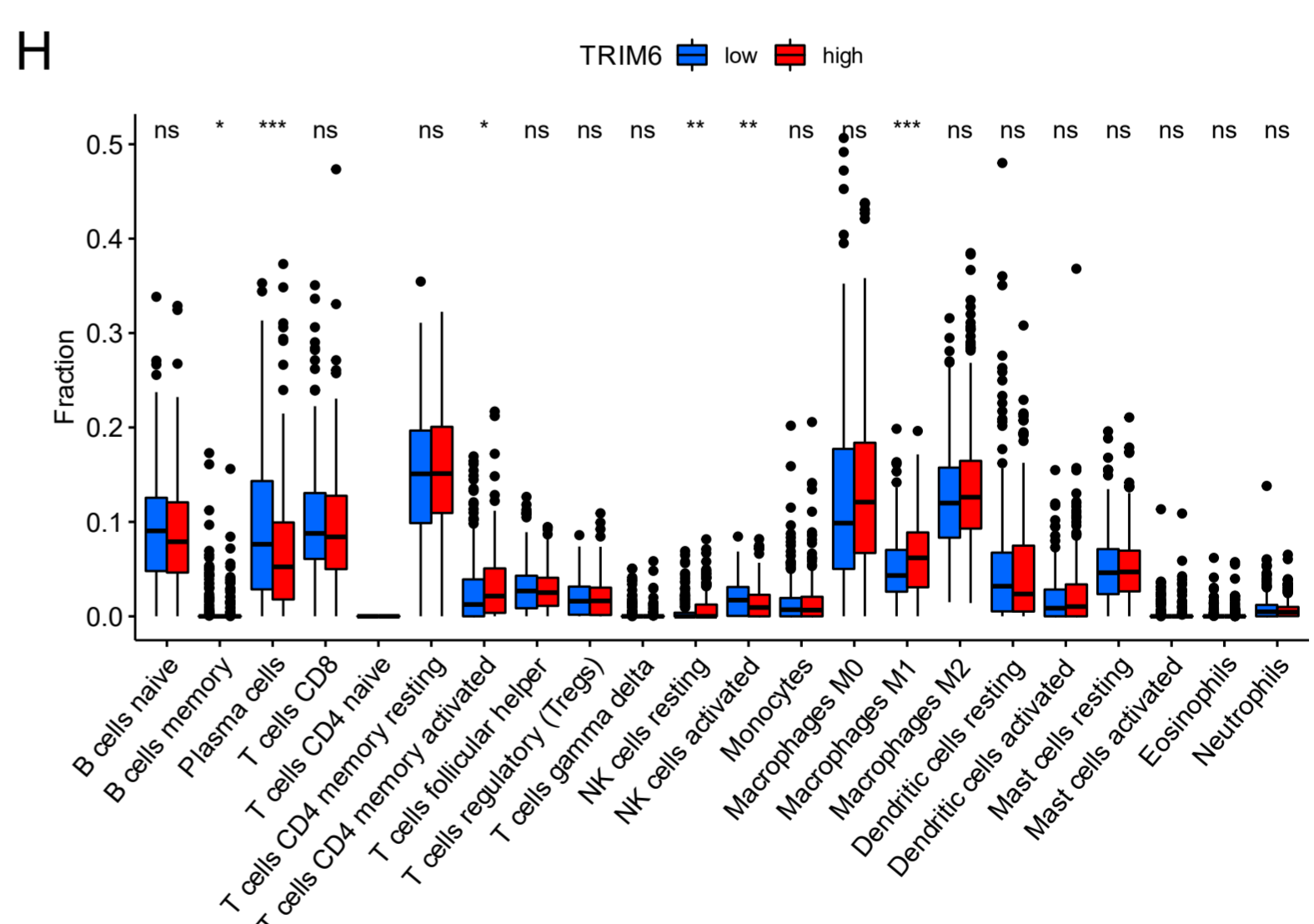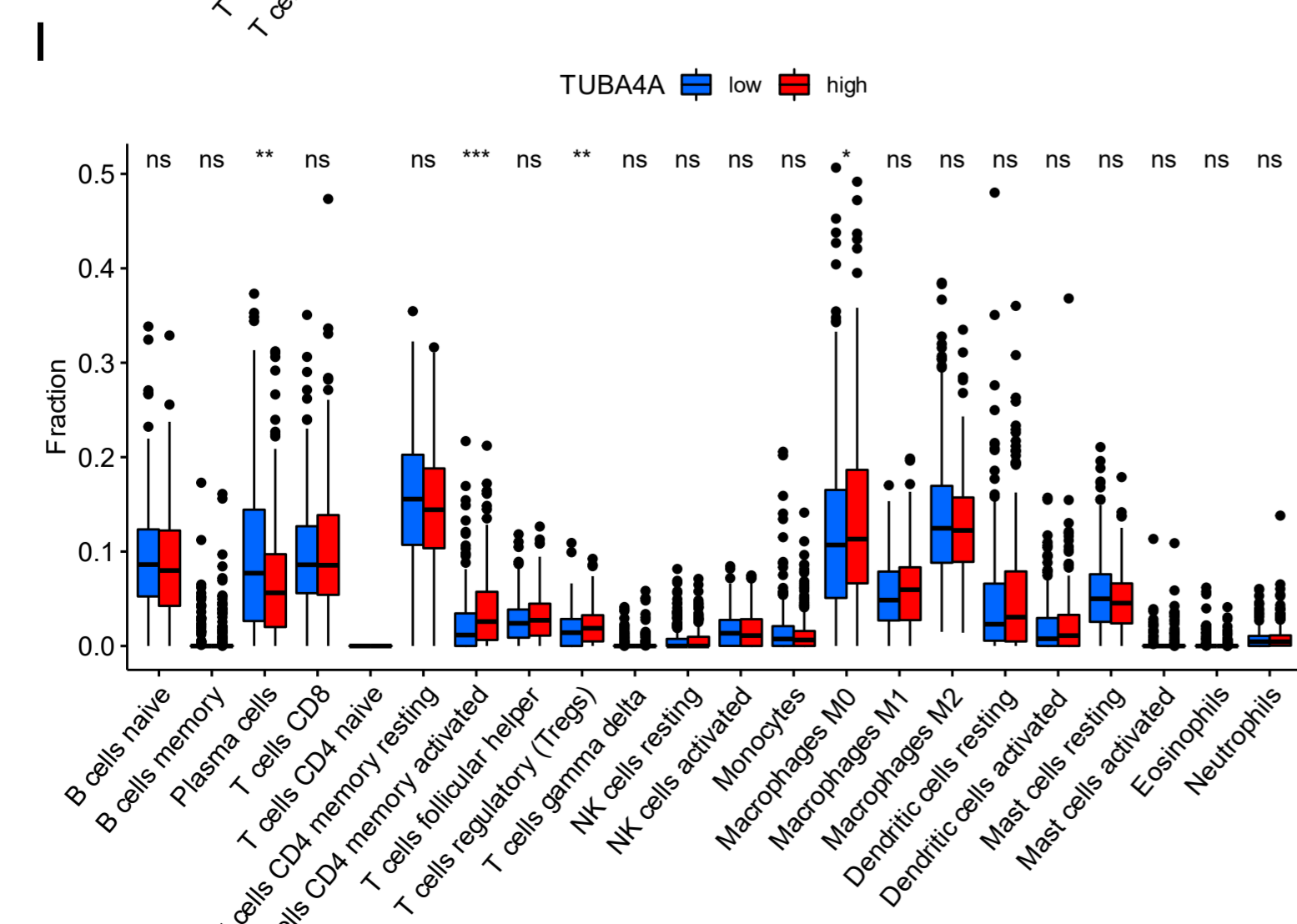

Supplement: Supplementary Materials — Figure S1 Analysis on the association between 9 signature genes and 22 immune cells. (A) ARRB1. (B) CCNB1. (C) CCT2. (D) PLK1. (E) PTTG1. (F) TRIM2. (G) TRIM58. (H) TRIM6. (I) TUBA4A. Table S1. All results of targeted therapy drugs prediction. [file 2524649.f1.zip › Figure S1.pdf]
